# The circular RNA PVT1/miR-203/HOXD3 pathway promotes the progression of human hepatocellular carcinoma

**DOI:** 10.1242/bio.043687

**Published:** 2019-09-24

**Authors:** Yiqing Zhu, Yan Liu, Bang Xiao, Hui Cai, Meng Liu, Liye Ma, Huirong Yin, Fang Wang

**Affiliations:** 1 Department of Medical Genetics, Second Military Medical University, Shanghai 200433, China; 2 Department of General Surgery, Changhai Hospital, Second Military Medical University, Shanghai, 200433, China; 3 Center of Reproductive Medicine, Changhai Hospital, Second Military Medical University, Shanghai, 200433, China; 4 Department of Clinical Genetics, Changhai Hospital, Second Military Medical University, Shanghai 200433, China

**Keywords:** CircPVT1, Hepatocellular carcinoma, MiR-203, HOXD3, Proliferation and migration

## Abstract

Accumulating evidence suggests that circular RNAs (circRNAs) play important roles in various physiological and pathological processes. In the present study, we explored the role of circRNA PVT1 in hepatocellular carcinoma (HCC). qRT-PCR was performed to detect the relative expression of circPVT1 in HCC tissues and cell lines. The oncogenic roles of circPVT1 in HCC were evaluated by cell counting kit-8 (CCK-8) assay, ethynyl deoxyuridine (EdU) incorporation assays, transwell assays, flow cytometry and *in vivo* xenograft growth. Furthermore, bioinformatics, luciferase reporter assays and rescue experiments were conducted to determine the underlying mechanism of circPVT1 in HCC. Enhanced circPVT1 expression was detected in HCC tissues, which was closely associated with poor prognosis of patients with HCC. Knockdown of circPVT1 decreased the proliferation and migration ability of HCC cell lines *in vitro*. Conversely, upregulation of circPVT1 improved the growth and migration in HCC cells. Mechanistically, we found that circPVT1 could bind directly to miR-203 and contributed to the initiation and progression of HCC by regulating miR-203/homebox D3 (HOXD3) pathway. In conclusion, our study reveals that circPVT1 participates in the progression of HCC through the miR-203/homeobox D3 (HOXD3) pathway and might represent a potential therapeutic target for HCC treatment.

## Abstract (precis)

**Summary:** Our research is the first to characterize the role of circPVT1 in the initiation and progression of HCC. CircPVT1 could be applied as a potential biomarker for the early screening of HCC.

## INTRODUCTION

Liver cancer is among the most common cancers worldwide, leading to approximately 600,000 deaths each year (Baffy et al., 2012). Hepatocellular carcinoma (HCC) accounts for more than half of all liver cancer and is closely related with chronic hepatitis B virus (HBV) infection (Lee et al., 2019). Although recent advances have been made in surgical treatment, effective therapy for HCC is still limited and the overall survival of HCC patients remains poor (Hlady and Robertson 2018). Early diagnosis and new therapeutic targets are two key points for successful intervention; therefore there is an urgent need to uncover the molecular mechanisms underlying the progression of HCC.

Circular RNAs (circRNAs) are a type of noncoding RNAs (ncRNAs) that are generated by circularization of introns or exons (Chen, 2016). Previously, circRNAs were regarded as byproducts of imperfect splicing without functional activity. However, recent progress in next-generation deep sequencing has reshaped our perspective of circRNAs (Li et al., 2018b). CircRNAs are widely expressed in all kingdoms of life, are evolutionary conserved and expressed in a cell-type or development stage specific manner. Moreover, increasing evidence shows that circRNAs play vital roles in the organization of cellular processes by acting as sponges for endogenous microRNAs (miRNAs) (Cadena and Hur, 2017; Han et al., 2018). Just like linear RNAs, certain circRNAs are reported to be disregulated in various kinds of cancer and usually indicate an unfavorable prognosis (Cui et al., 2018). For example, the expression level of circPVRL3 was decreased in gastric cancer and was associated with unfavorable overall survival (Sun et al., 2018). Circ-ANAPC7 participated in the progression of acute myeloid leukemia by binding to miR-181 (Chen et al., 2018). Thus, studies on circRNAs may hold the potential for better understanding of carcinogenesis and progression. However, the exact role of circRNAs in HCC remains elusive.

CricPVT1 is a newly identified circRNA that is located at chr8: 128902834-128903244, a known cancer susceptibility region (Panda et al., 2017). It is derived from the third exon of lncRNA-PVT1, which is identified as an oncogene (Riquelme et al., 2014; Yang et al., 2014; Zeng et al., 2015). A previous study showed that circPVT1 was upregulated in gastric cancer and could be a potential prognostic marker (Chen et al., 2017). Nevertheless, the expression patterns and biological functions of circPVT1 in HCC are largely unknown. In the present study, we detected the relative expression of circPVT1 in HCC tissues and cells. *In vitro* and *in vivo* studies were conducted to elucidate the biological effects of circPVT1 on HCC cell growth and migration. Furthermore, we performed luciferase reporter assays and rescue experiments to reveal the underlying mechanisms. We discovered that circPVT1 participated in the progression of HCC through regulating miR-203/homeobox D3 (HOXD3) pathway.

## RESULTS

### CircPVT1 expression is significantly upregulated in both HCC tissues and cell lines and suggests poor prognosis in patients with HCC

**Fig. 1.**
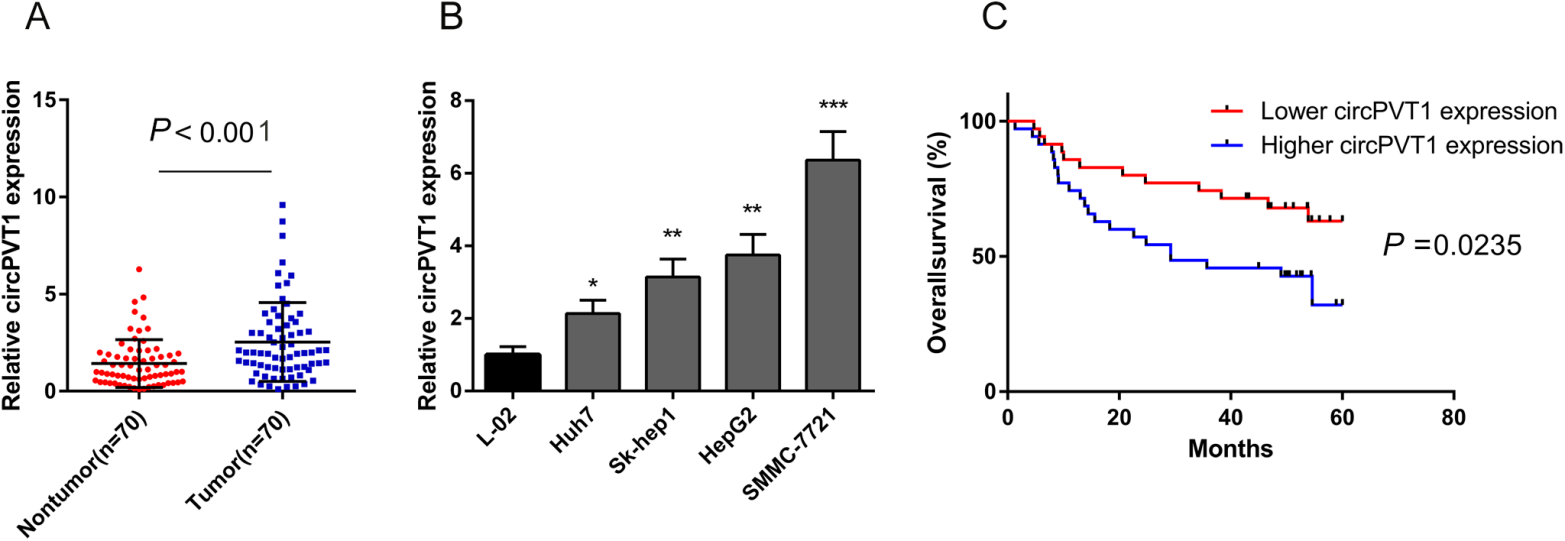
**Relative expression of circPVT1 in HCC** **tissues and HCC cell lines and its prognosis potential in HCC patients.** (A) CircPVT1 expression was detected in 70 pairs of HCC tissues and adjacent non-tumor tissues by qRT-PCR, Wilcoxon signed-rank test. (B) Relative expression of circPVT1 in HCC cell lines and normal liver cells was measured by qRT-PCR. (C) The relationship between relative expression of circPVT1 and HCC patients' overall survival was analyzed by log-rank test and Kaplan–Meier method. *P<0.05, **P<0.01, ***P<0.001. Mean±s.e., *n*=3 in B.

qRT-PCR was conducted to detect the relative expression of circPVT1 in 70 pairs of HCC clinical tissues. The data revealed that circPVT1 was significantly upregulated in HCC tissues compared with the paired non-tumor tissues (Fig. 1A). In addition, circPVT1 expression in HCC and normal liver cell lines were also detected. Compared with non-cancerous L-02 cells, circPVT1 expression was significantly upregulated in all HCC cell lines (Fig. 1B). We further characterized the clinical significance of circPVT1 expression in HCC tissues. The results showed that the expression of circPVT1 was closely associated with overall survival (Fig. 1C), lymph node metastasis and tumor-node-metastasis (TNM) stages (Table 1). These suggested that circPVT1 might contribute to the initiation and progression of HCC.

**Table 1.**
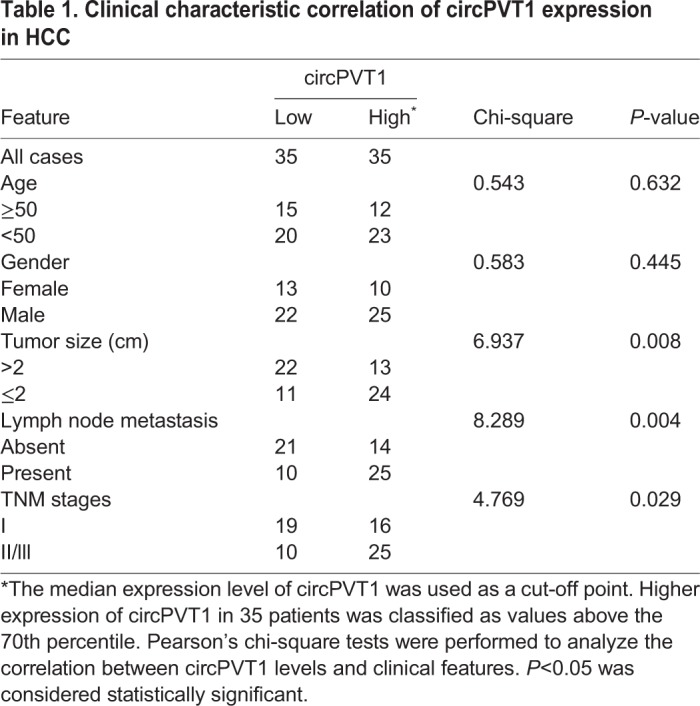
**Clinical characteristic correlation of circPVT1 expression in HCC**

### CircPVT1 promotes the proliferation and migration of HCC cells *in vitro*

**Fig. 2.**
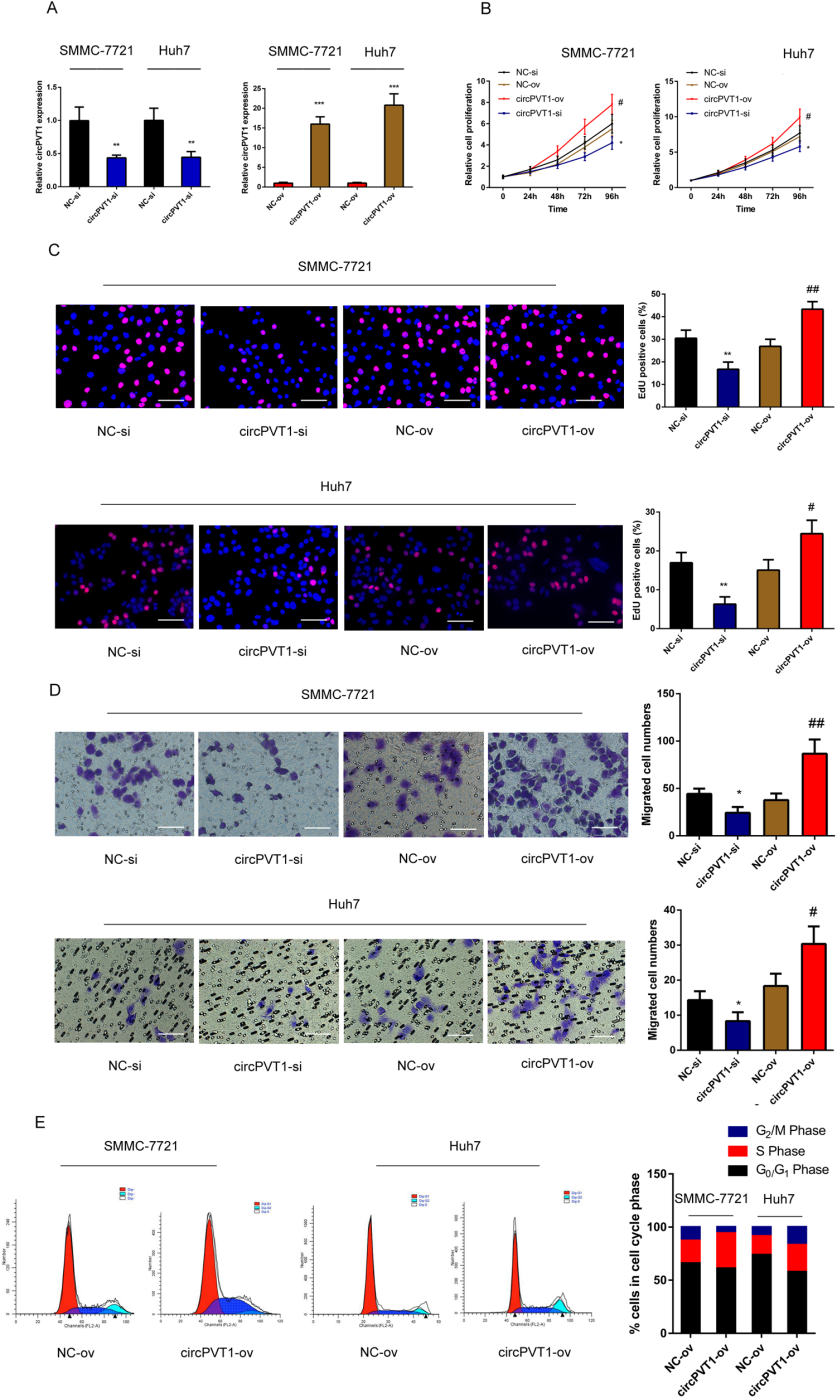
**CircPVT1 promotes proliferation and migration of HCC cells.** (A) CircPVT1 expression was detected in SMMC-7721 and Huh7 cells by qRT-PCR. (B) The effects of circPVT1 on proliferation of SMMC-7721 and HCC cells were measured by CCK-8 assay. (C) The effects of circPVT1 on proliferation of SMMC-7721 and Huh7 cells were measured by EdU assay. Nuclei  represented in blue, and EdU-positive cells are marked by pink. (D) The migration ability of SMMC-7721 and Huh7 cells with circPVT1 overexpressed or depressed was detected by transwell assay. (E) Cell cycle was measured by flow cytometry. NC-si, negative control of cells with circPVT1 knockdown; NC-ov, negative control of cells with circPVT1 overexpressed; circPVT1-si, cells treated with siRNA target circPVT1; circPVT1-ov, cells with circPVT1 overexpressed. **P<0.01, ***P<0.001 compared with NC-ov in A, **P<0.01, ***P<0.001 compared with NC-si in B; *P<0.05, **P<0.01 compared with NC-si in C,D; ^#^*P*<0.05, ^##^*P*<0.01 compared with NC-ov in C,D. Mean±s.e., *n*=3. Scale bars: 100 µm.

To investigate the biological function of circPVT1 in HCC, SMMC-7721 and Huh7 cell lines were adopted for the following experiments. CricPVT1 was either overexpressed or downregulated in SMMC-7721 and Huh7 cell lines, respectively, and the efficiency was confirmed by qRT-PCR (Fig. 2A). Meanwhile, overexpression of circPVT1 does not influence the expression of lncRNA-PVT1 in HCC cell lines (Fig. S1). Cell Counting Kit-8 (CCK-8) and ethynyl deoxyuridine incorporation (EdU) assays were conducted to evaluate the role of circPVT1 in HCC cell growth. As shown in Fig. 2B and C, circPVT1 knockdown dramatically inhibited the proliferation of SMMC-7721 and Huh7 cells. Conversely, overexpression of circPVT1 resulted in an increase in SMMC-7721 and Huh7 cells growth (Fig. 2B,C). Furthermore, transwell assays revealed that silencing circPVT1 markedly decreased the migration ability of SMMC-7721 and Huh7 cells (Fig. 2D,E), while the cell migration ability of SMMC-7721 and Huh7 cells was markedly enhanced by circPVT1 overexpression (Fig. 2D). Moreover, cell cycle analysis showed that overexpression of circPVT1 resulted in increased cell distribution in the S phase (Fig. 2E).

### Overexpression of circPVT1 increased HCC cell growth *in vivo*

**Fig. 3.**
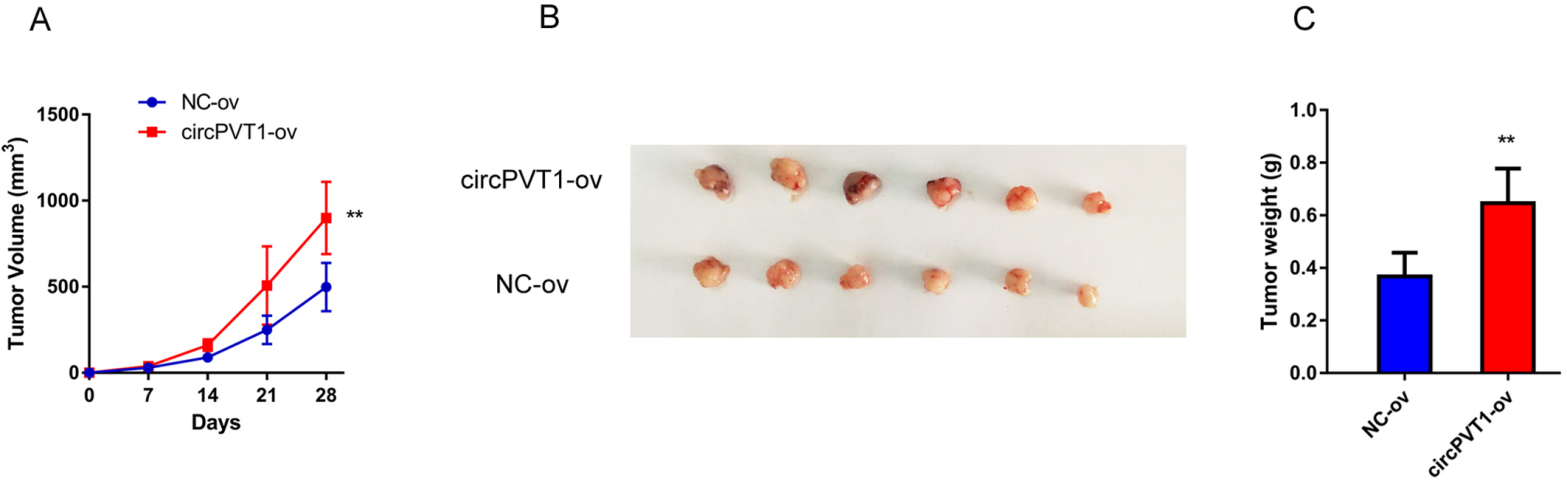
**CircPVT1 promotes HCC cell growth *in vivo*.** (A) Tumor volume was measured every 7 days. (B) The tumors excised from the nude mice. (C) The tumors were weighed after excision. NC-ov, negative control of cells with circPVT1 overexpressed; circPVT1-ov, cells with circPVT1 overexpressed. **P<0.01 compared with NC-si. Mean±s.e., *n*=6/group.

To further confirm the proliferative effects of circPVT1 on HCC cells *in vivo*, we injected Huh7 cells subcutaneously into athymic nude mice. Consistent with the *in vitro* results, the overall growth of Huh7 cells with circPVT1 overexpression was increased (Fig. 3A). Moreover, the volumes of the tumors derived from circPVT1-overexpressing xenografts were larger than those of the negative control (Fig. 3B,C). These results demonstrated that overexpression of circPVT1 promoted the growth of HCC cells *in vivo*.

### miR-203 is the direct target of circPVT1

**Fig. 4.**
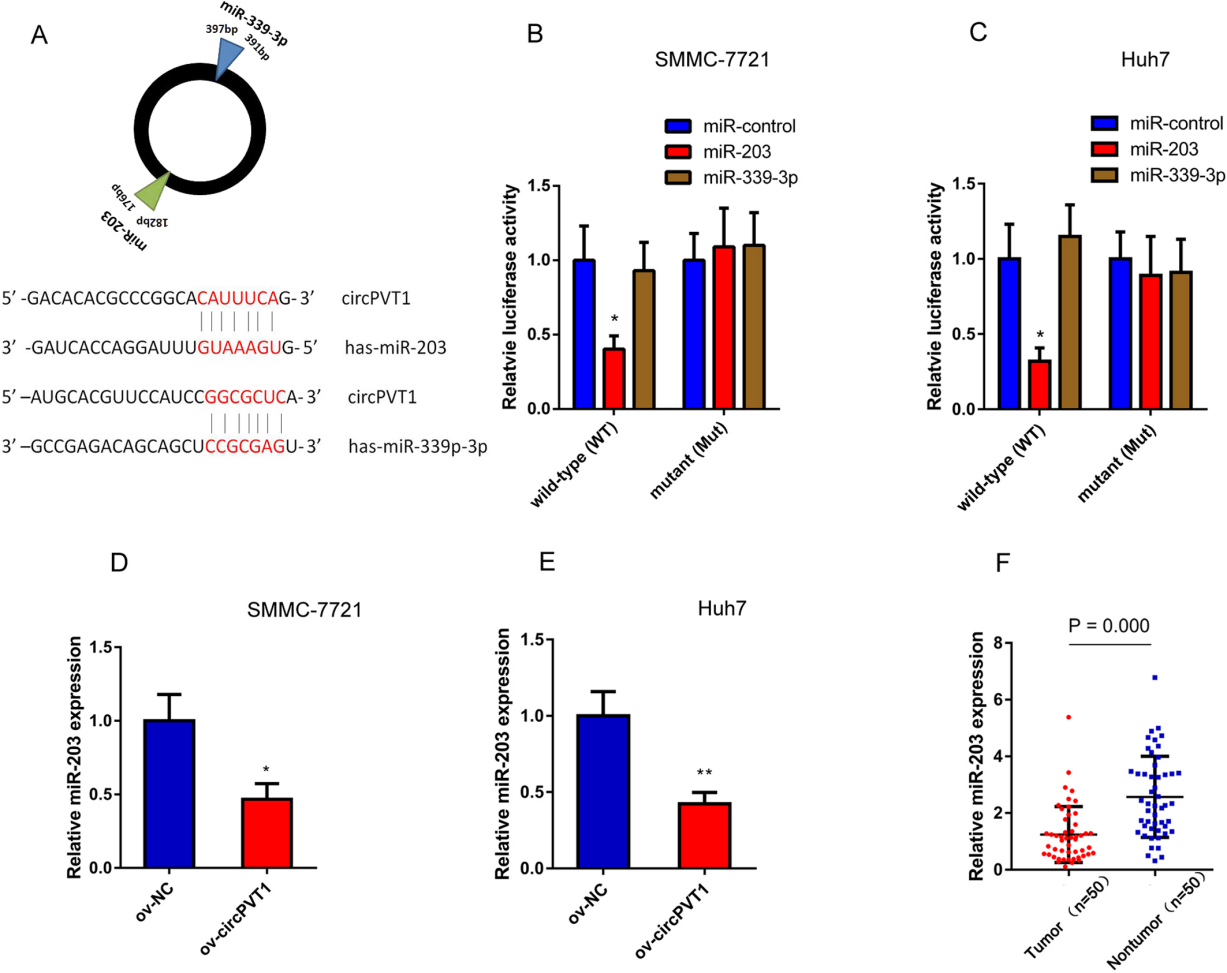
**CircPVT1 directly binds to miR-203.** (A) The predicted binding sites of circPVT1 to miR-203 and miR-339p-3b. (B,C) The luciferase activity of circPVT1 or circPVT1-Mut in Huh7 and SMMC-7721 cells co-transfected with miRNA mimics. (D,E) Relative expression of miR-203 in SMMC-7721 and Huh7 cells with circPVT1 overexpression. (F) Relative expression of miR-203 in tumor and non-tumor tissues from HCC patients was detected by qRT-PCR. ov-NC, negative control of cells with circPVT1 overexpressed; ov-circPVT1, cells with circPVT1 overexpressed. *P<0.05, **P<0.01 compared with ov-NC. Mean±s.e., *n*=3.

Accumulating studies indicate that circRNAs might take part in regulating endogenous RNAs through binding to functional miRNAs. Thus, we hypothesized that circPVT1 could contribute to tumorigenesis of HCC by targeting miRNAs. To identify candidate targets of circPVT1, a public database (https://circinteractome.nia.nih.gov/) was used. The results showed that tumor suppressors, miR-339-3p and miR-203, have binding sites for circPVT1 (Fig. 4A). To determine whether circPVT1 binds to miR-339-3p and miR-203 directly, luciferase assays were performed. Compared with the control, miR-203 significantly reduced the luciferase reporter activity in Huh7 and SMMC-721 cells (Fig. 4B,C). Meanwhile, overexpression of circPVT1 significantly inhibited miR-203 expression in both Huh7 and SMMC-7721 cells (Fig. 4D,E). Finally, we detected the expression of miR-203 in 50 paired tumor and non-tumor tissues from HCC patients by qRT-PCR. miR-203 was downregulated in HCC tissue samples (Fig. 4F) and was negatively correlated with circPVT1 expression, indicating that circPVT1 could sponge and regulate miR-203.

### CircPVT1 contributes to the progression and migration of HCC cells through miR-203/HOXD3 pathway

**Fig. 5.**
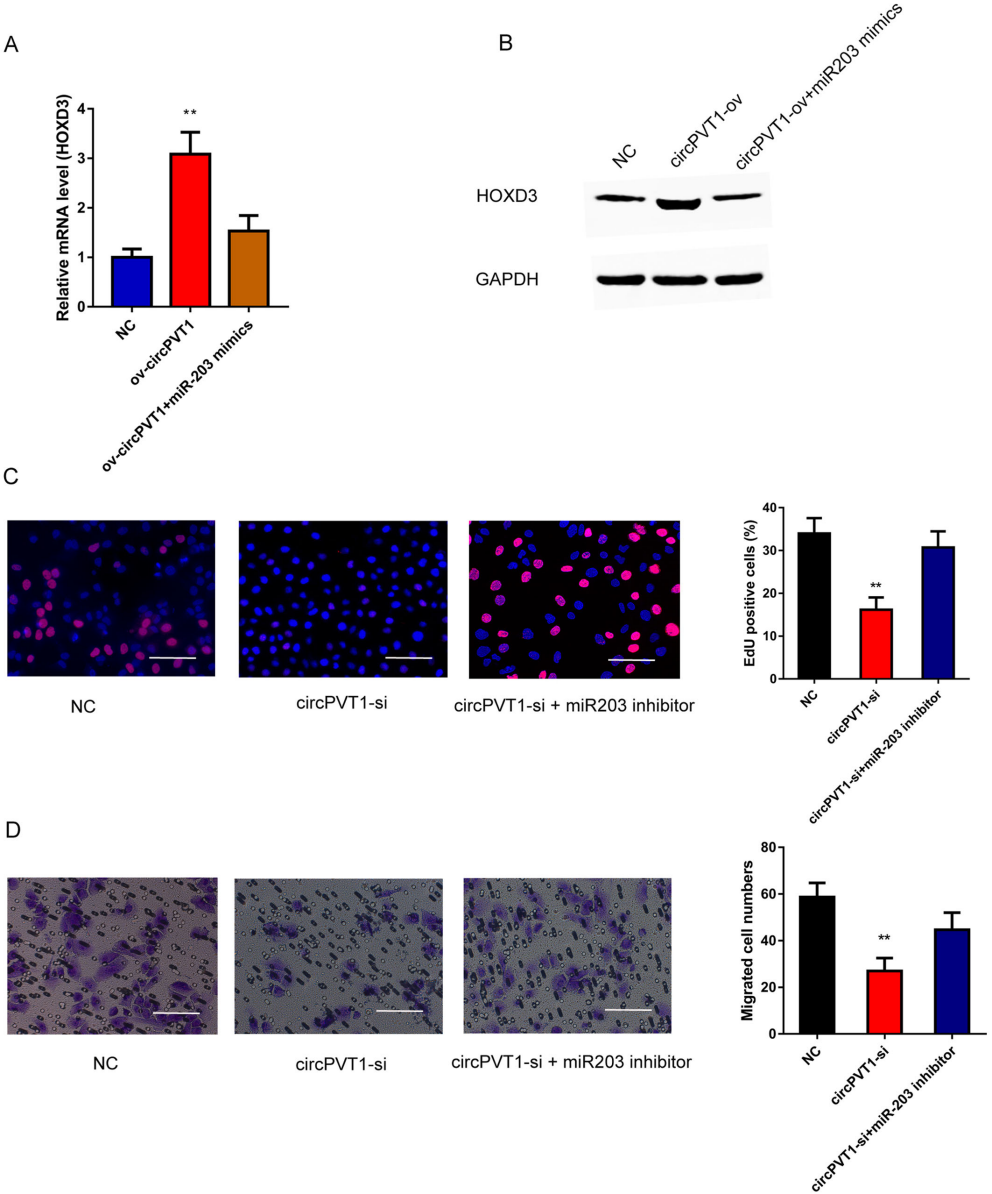
**circPVT1 promotes HCC cell proliferation and migration by binding to miR-203 and negative regulates HOXD3.** (A,B) qRT-PCR and western blot was performed to detect relative expression of HOXD3 in Huh7 cells after transfection. (C) EdU assays were performed to detect the proliferation ability of Huh7 cells after transfection. (D) Transwell assays were performed to detect the migration ability of Huh7 cells after transfection. NC, negative control; circPVT1-si, cells with circPVT1 knockdown; circPVT1-si+miR-203 inhibitor, cells with circPVT1 knockdown and treated with miR-203 inhibitor. **P<0.01. Mean±s.e., *n*=3. Scale bars: 100 µm.

Previous studies have reported that HOXD3, which acts as an oncogene in HCC, is a direct target of miR-203. In our research, qRT-PCR and western blot analysis revealed that circPVT1 overexpression induced the upregulation of HOXD3 in Huh7 cells, while miR-203 mimics inhibited HOXD3 expression (Fig. 5A,B). EdU assays revealed that inhibition of miR-203 could rescue the circPVT1 silencing-mediated reduced Huh7 cell growth (Fig. 5C). Moreover, transwell assays showed that treatment of miR-203 inhibitor rescued the downregulation of migratory ability induced by circPVT1 knockdown (Fig. 5D). Taken together, these results suggest that the functional role of circPVT1 in HCC might depend on its direct binding to miR-203 and regulation of HOXD3 expression.

## DISCUSSION

As an aggressive malignancy, HCC is characterized by high recurrence rate and mortality. With accumulating deep sequencing results, substantial amounts of ncRNAs are reported to be dysregulated in HCC (Liu et al., 2019; L'Hermitte et al., 2019). However, few studies have explored the role of circRNAs in HCC. More and more evidence has revealed that circRNAs are involved in various physiological activities and disease pathogeneses (Croce, 2016). CircRNA ITCH plays an anti-oncogenic role in glioma (Li et al., 2018a). Circ-ITCH significantly inhibits the proliferation and migration of glioma cells through sponging miR-214. Upregulation of circRNA_0023642 is detected in gastric cancer (Zhou et al., 2018). CircRNA_0023642 contributes to the progression of gastric cancer by promoting epithelial-mesenchymal transition. CircPRKCI is overexpressed in lung adenocarcinoma and promotes tumorigenesis through acting as sponge for miR-589 and miR-545 (Qiu et al., 2018). Meanwhile, given their high stability and conserved expression pattern, circRNAs are promising therapeutic targets and potential diagnosis markers for cancer. However, HCC progression-related circRNAs are rarely reported.

In the present study, we explored the role of circPVT1 in the progression of HCC. CircPVT1 is generated by exon 3 circularization from lncRNA-PVT1, which is characterized as an oncogene in several types of cancer. Our previous study demonstrated that lncRNA-PVT1 promoted proliferation and stem cell-like properties of HCC cells by enhancing NOP2 nucleolar protein stability (Wang et al., 2014). The present study revealed that, compared with normal tissues, the relative expression of circPVT1 was significantly upregulated in HCC. Meanwhile, circPVT1 expression was more enhanced in HCC cells than that in normal liver cells. Clinical analysis demonstrated that expression of circPVT1 was closely associated with overall survival, lymph node metastasis, and TNM stages, which suggested the clinical significance of circPVT1. Moreover, function experiments showed that circPVT1 knockdown significantly dampened the proliferation and migration ability of HCC cells. However, upregulation of circPVT1 had the opposite effect. To sum up, our results indicated that circPVT1 had oncogenic effects on HCC and might offer new clues for HCC treatment.

As competing endogenous RNA (ceRNA) hypothesis proposes, ncRNAs can act as miRNA ‘sponges’ that competitively attract miRNAs and sequester miRNAs from their target mRNAs (Zhang et al., 2018). miRNAs are small non-coding RNAs and regulate mRNA expression through RNA silencing (O'Brien et al., 2018). Thus, dysregulated ncRNA-miRNA networks are associated with the development and progression of various diseases. Recently, emerging evidence has shown that circRNAs can also bind miRNA by complementary base pairing and regulate the downstream genes of miRNA. For instance, the expression of circ_0016760 was increased in non-small cell lung cancer (NSCLC) tissues and regulated GAGE1 expression by sponging miR-1287 (Li et al., 2018c). Liu reported that circHIPK3 acted as a sponge for miR-193a and participated in the progression of age-related cataracts (Liu et al., 2018). In our study, bioinformatic analysis showed that miR-203 and miR-339-3p were potential targets of circPVT1. Luciferase reporter assay revealed that circPVT1 directly contacted with miR-203. Moreover, miR-203 expression could be regulated by circPVT1 in HCC cells and was downregulated in HCC tissue samples.

It has been widely acknowledged that miR-203 acts as a tumor suppressor and participates in the initiation and maintenance of HCC (Zhang et al., 2017). HOXD3 was the downstream target of miR-203 and contributed to the growth and migration of HCC cells (Wang et al., 2016; Wang et al., 2018). Thus, we further detected the role of miR-203 in circPVT1-mediated progression of HCC. In our study, results showed that circPVT1 overexpression increased the expression of HOXD3 and that treatment with miR-203 mimics had the opposite effect. Rescue experiments showed that treatment with miR-203 inhibitors rescued the decreased proliferation and migration ability of HCC cells induced by knockdown of circPVT1. Collectively, these results indicated that circPVT1 is involved in the development of HCC by regulating the miR-203/HOXD3 pathway.

In conclusion, our study demonstrated that circPVT1 was upregulated in HCC tissues and cells and was closely associated with clinical characteristics of HCC patients. Functional experiments showed that circPVT1 worked as an oncogene to promote HCC growth and migration. Further mechanistic studies indicated that circPVT1 bound competitively to miR-203 and that the miR-203/HOXD3 pathway contributed to the oncogenic role of circPVT1. The circPVT1/miR-203/HOXD3 regulatory axis might offer a new strategy for HCC diagnosis and intervention.

## MATERIALS AND METHODS

### Tissue samples

Seventy paired HCC tissues and adjacent non-tumor tissues were collected from the Eastern Hepatobiliary Surgery Hospital (Shanghai, China). The clinicopathologic characteristics of all the HCC patients were documented and summarized in Table 1. This study was conducted with the approval from the Committees for Ethical Review of Second Military Medical (Shanghai, China).

### Cell cultures

The human HCC cell lines (Huh7, Sk-hep1, SMMC-7721 and HepG2) and normal human liver cells (L-02) were purchased from Chinese Academy of Science Cell Bank. The cells were cultured in high glucose DMEM medium (Invitrogen) supplemented with 5–10% fetal bovine serum (Gibco, USA) and incubated with 5% CO_2_ at 37°C.

### Cell transfection

Small interfering RNA (siRNA) against circPVT1 (5′-CUGUCAGCUGCAUGGAGCUUCGU-3′), miR-203 mimic, miR-339-3p mimic and miR-203 inhibitor (purchased from GenePharm, Shanghai, China) were transfected by Lipofectamine 3000 (Thermo Fisher Scientific).

For circPVT1 overexpression, the genomic region of circPVT1 and its franking introns was subcloned into a pcDNA3.1 vector and transfected into liver cancer cells with Lipofectamine 3000 (Thermo Fisher Scientific).

### RNA extraction and quantitative real-time PCR

Total RNAs were isolated with RNAiso Plus (Takara, China). Then complementary DNA was synthesized by PrimeScript™ RT Master Mix (Takara, China). qPCR was conducted using SYBR Green in StepOneTM Real-Time PCR system (Applied Biosystems). GAPDH was used as endogenous control here. The sequences of primers are listed here: for circPVT1, 5′-GGTTCCACCAGCGTTATTC-3′ (forward) and 5′-CAACTTCCTTTGGGTCTCC-3′ (reverse); for lncRNA-PVT1, 5'-TTCAGCACTCTGGACGGACTT-3′ (forward) and 5′-TATGGCATGGGCAGGGTAG-3′ (reverse); for GAPDH, 5'-GGAGCGAGATCCCTCCAAAAT-3′ (forward) and 5′-GGCTGTTGTCATACTTCTCATGG-3′ (reverse); for miR-203, 5′-GTCGTACCAGTGCAGGGTCCGAGGTATTCGCACTGGATACGACCTAGT-3′ (forward) and 5′-GCCCGTGAAATGTTTAGGACCAC-3′ (reverse). The expression levels of the target were calculated by the 2^−ΔΔCt^ method.

### Cell proliferation assays

The proliferation ability of cells was evaluated by CCK-8 assay and EdU assay. For CCK-8 assay, 3×10^3^ cells were seeded in the well of a 96-well plate and were incubated for an appropriate length of time (12, 24, 48, 72 h). The obtained cells then had 10 µl CCK-8 solution added and we measured the absorbance at 450 nm with a microplate reader (Biotek, Winooski, VT). EdU assays were performed using an EdU kit (Ribobio, China) following the instruction. Zeiss axiophot photomicroscope was adopted here to snap the results (Carl Zeiss, Germany) and Image-Pro plus 6.0 software was used for further analysis.

### Cell migration assays

The migration ability of HCC cells were detected by transwell assay. In short, about 4×10^4^ cells were planted in the inner chamber of transwell (Millipore) with basic DMEM medium and the lower chamber was filled with fetal serum supplemented medium. Cells were incubated for 24 h. With cells on the inner surface of the chamber wiped off, the remaining cells were fixed for 20 min, stained, photographed and counted.

### Cell cycle analysis

Flow cytometry was performed to detect the cell cycle. In short, cells were collected and stained with Propidium Iodide (PI). Fluorescence-activated cell sorting (FACS) analysis was used to analyze cell cycles. The proportion of cells in each phase, G_0_–G_1_, S and G_2_–M, were counted and compared.

### Luciferase reporter assay

The circular RNA Interactome database was adopted here to predict the interaction between circPVT1 and miRNAs (https://circinteractome.nia.nih.gov). For luciferase reporter assays, the coding region of circPVT1 was subcloned into a pmirGLO vector which was recorded as pmirGLO-circPVT1-WT. Meanwhile, the coding region of circPVT1 with binding sites for miR-339-3p and miR-203-mutated was subcloned into a pmirGLO vector which was recorded as pmirGLO-circPVT1-MUT. Then, Huh7 cells and SMMC-7721 cells were transfected with pmirGLO-circPVT1-WT vector+miR-203 mimics, pmirGLO-circPVT1-WT+miR-339-3p mimics, pmirGLO-circPVT1-WT vector+mimics-NC, pmirGLO-circPVT1-MUT vector+miR-203 mimics, pmirGLO-circPVT1-MUT vector+mimics-NC, or pmirGLO-circPVT1-MUT+miR-339-3p mimics by Lipofectamine 3000. 48 h after transfection, luciferase reporter assay (Promega) was performed according to the manufacturer's instructions. In the experiment, activities of Firefly luciferases and Renilla luciferases were measured sequentially from a single sample. Activities of Renilla luciferases were used to normalize Firefly luciferases activities.

### Western blot analysis

The following primary antibodies were used to conduct western blot analysis: anti-HOXD3 (1:5000, ab22840, Abcam) and anti-GAPDH (1:5000, ab9485, Abcam). Briefly, cells lysates were harvested and boiled in SDS-PAGE loading buffer. Equal amounts of cell lysates were separated with 10% SDS-PAGE gel and were transferred onto nitrocellulose membranes. We then incubated the membranes with primary antibodies overnight at 4°C. After being eluted with PBST three times, the membranes were incubated with goat anti-mouse IgG. The signals were collected with Odyssey infrared imaging system (Li-Cor; USA).

### Animal studies

Male athymic BALB/c mice (5-week-old) were purchased from the Shanghai Experimental Animal Center (Shanghai, China) and kept under speciﬁc pathogen-free conditions for further studies. Each mouse limb was injected with 3×10^6^ cells subcutaneously and the mice were kept for 1 month. The tumor size was monitored and calculated with the following function: volume=0.4×length×width^2^. The above studies were conducted with approval from the Animal Care and Use Committee of Second Military Medical University.

### Statistical analysis

SPSS software (version 17.0) was applied for all the statistical analyses. One-way analysis of variance, the chi-square test, Mann–Whitney test and the two-tailed Student's *t*-tests were used as appropriate. Patients' survival was evaluated by Kaplan–Meier method tests. All experiments were performed in triplicate. *P*<0.05 was defined as statistically significant.

## Supplementary Material

Supplementary information
