# Supplementary material for: The circular RNA PVT1/miR-203/HOXD3 pathway promotes the progression of human hepatocellular carcinoma
Source: Biol Open. 2019 Sep 24;8(9):bio043687. doi: 10.1242/bio.043687 (PMC6777361; doi:10.1242/bio.043687)
Supplement: Supplementary information [file biolopen-8-043687-s1.pdf]

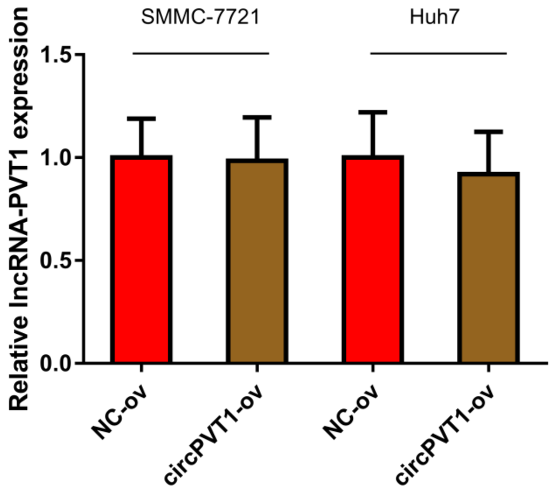

Figure S1. lncRNA-PVT1 expression in HCC cell lines.

(A) LncRNA-PVT1 expression was detected in SMMC-7721 and Huh7 cells by qRT-PCR.

Biology Open • Supplementary information
